# Lotus leaf flavonoids induce apoptosis of human lung cancer A549 cells through the ROS/p38 MAPK pathway

**DOI:** 10.1186/s40659-021-00330-w

**Published:** 2021-03-02

**Authors:** Xiang-Bo Jia, Quan Zhang, Lei Xu, Wen-Jian Yao, Li Wei

**Affiliations:** Department of Thoracic Surgery, Zhengzhou Key Laboratory of Surgical Treatment for End-Stage Lung Diseases, Henan Provincial People’s Hospital, People’s Hospital of Zhengzhou University, Zhengzhou, 450003 Henan China

**Keywords:** Human lung cancer A549 cells, Flavonoid, Lotus leaf, C57BL/6J mice, mRNA ROS/p38 MAPK pathway

## Abstract

**Background:**

Leaves of the natural plant lotus are used in traditional Chinese medicine and tea production. They are rich in flavonoids.

**Methods:**

In this study, lotus leaf flavonoids (LLF) were applied to human lung cancer A549 cells and human small cell lung cancer cells H446 in vitro to verify the effect of LLF on apoptosis in these cells through the ROS/p38 MAPK pathway.

**Results:**

LLF had no toxic effect on normal cells at concentrations up to 500 µg/mL, but could significantly inhibit the proliferation of A549 cells and H446 cells. Flow cytometry showed that LLF could induce growth in A549 cells. We also found that LLF could increase ROS and MDA levels, and decrease SOD activity in A549 cells. Furthermore, qRT-PCR and western blot analyses showed that LLF could upregulate the expression of p38 MAPK (p-p38 MAPK), caspase-3, caspase-9, cleaved caspase-3, cleaved caspase-9 and Bax and downregulate the expression of Cu/Zn SOD, CAT, Nrf2, NQO1, HO-1, and Bcl-2 in A549 cells. Results of HPLC showed that LLF mainly contain five active substances: kaempferitrin, hyperoside, astragalin, phloridzin, and quercetin. The apoptosis-inducing effect of LLF on A549 cells came from these naturally active compounds.

**Conclusions:**

We have shown in this study that LLF is a bioactive substance that can induce apoptosis in A549 cells in vitro, and merits further research and development.

## Background

Lung cancer is one of the most dangerous malignant tumors in humans. The world’s incidence and death rates are about 1.86 million and 1.6 million per year, respectively. In some countries, death rate of lung cancer is higher than any other type of cancer [1]. Because most lung cancer patients are already in the late stage by the time of diagnosis, the opportunity for surgical treatment has already been lost, so the main clinical treatments are radiotherapy and chemotherapy [2]. Although most lung cancer patients respond to the initial treatment, the side effects of chemotherapy are severe and drug resistance is easily acquired. The effect of second-line chemotherapy is not as good as the first [3]. It is therefore a research priority to find safe and effective natural bioactive substances for lung cancer prevention and adjuvant treatment.

Two main kinds of lotus (*Nelumbo nucifera*, Gaertn) plants are recognized, one is the American lotus in the northeast part of America; the other is the Chinese lotus, centered in Asia [4]. At present, Chinese lotus is widely distributed in Hunan, Hubei, Zhejiang, Jiangsu, and other regions in China [5]. According to traditional Chinese medicine, lotus leaves are mainly used to clear away heat, thirst, and dampness, raise hair, clear Yang, cool blood, and stop bleeding, diarrhea, and vomiting, and prevent spleen deficiency and other diseases [6–8]. The main active components of lotus leaves are flavonoids and alkaloids. At present, there is extensive research activity on the effects of lotus leaves flavonoids on lowering blood lipids, treatment and prevention of allergy, cancer, and cardiovascular diseases, as well as its activity as anti-aging, bacteriostatic, and anti-oxidation agent [9–12]. Because of its high bioactivity and low cost, flavonoids from lotus leaves became an important target for research.

p38 MAPK and c-Jun N-terminal kinase (JNK) are also known as stress-activated protein kinases (SAPKs) as they are often activated by some environmental stress stimulus and cytokines that lead to inflammation. Excessive inflammation can cause many diseases, including cancer [13]. The MAPK pathway, especially p38 MAPK, has become an important potential target in inflammation and cancer prevention and treatment [14]. Oxidative stress can induce receptor-dependent apoptosis and damage the mitochondria in normal cells. Mitochondrial dysfunction will further increase ROS accumulation and activate the p38 MAPK pathway [15]. The relationship between ROS/p38 MAPK pathway, inflammation, and tumor growth needs further research, and so do the biological effects of active natural substances on this pathway.

There are many studies on the anticancer effect of plant flavonoids. Based on the knowledge derived from previous reports, this study aimed to further elucidate the effects of lotus leaf flavonoids (LLF) on apoptosis of human lung cancer A549 cells, explore the molecular pathways through which LLF exert their inhibitory effects on the growth of these cells, and provided theoretical basis for future LLF application.

## Materials and methods

### Extraction of lotus leaf flavonoids

Lotus leaves (200 g, Anhui Xile Garden Food Co., Ltd., Anhui, China) were crushed and placed inside a 5-L beaker, to which 4 L of 70% ethanol were added, based on a lotus leaves to 99% ethanol ratio of 1:20 (w/v for lotus leaves and ethanol). The beaker was sealed with plastic film and placed in a water bath at 60 °C for 3 h. After cooling, the mixture was filtered, and the crude extract of LLF was obtained. FL-3 macroporous resin was loaded into the glass column, and the crude extract of LLF was slowly poured over the macroporous resin. The filtrate that passed through the resin was discarded, and the resin was eluted with 70% ethanol until it became colorless. The eluted solution was collected, steamed, and ethanol in the solution was removed by rotary evaporation. The extracted LLF were freeze-dried to obtain the purified flavonoids powder.

### HPLC assay

To make the reference stock solution, the standard (20 mg) was precisely weighed and then well shaken with 2 mL of chromatographic grade methanol till it was completely dissolved. LLF were tested under the following liquid chromatography (Ultimate3000; Thermo Fisher Scientific, Inc., Waltham, MA, USA) conditions: the chromatographic column was Agilent zorbax SB-C18 (5 µm, 4.6 × 250 mm), 0.1% glacial acetic acid was used as mobile phase B, pure acetonitrile was used as mobile phase C, column temperature was controlled at 35 °C, flow rate was set at 0.5 mL/min, detection wavelength was 360 nm, and injection volume was 10 µL. The gradient elution conditions of the mobile phase are shown in Table 1. The content of each component was calculated according to the standard curve (Table 2).

**Table 1:** Gradient elution conditions of mobile phase

Time (min)	Current speed (mL/min)	%B	%C
− 10.000	0.500	88.0	12.0
0.000	0.500	88.0	12.0
30.000	0.500	60.0	40.0
35.000	0.500	0.0	100.0
40.000	0.500	0.0	100.0

**Table 2:** Linear regression equation of compounds

Compound	Linear equation	R^2^
Kaempferitrin	y = 8.1449x + 3.7423	0.9844
Hyperoside	y = 3.1871x + 9.5149	0.9897
Astragalin	y = 11.326x + 0.8521	0.9994
Phloridzin	y = 0.9852x + 0.3022	0.9843
Quercetin	y = 10.369x − 3.0987	0.9990

### MTT assay

Human lung cancer A549 cells, human small cell lung cancer cells H446 and BEAS-2B lung bronchus epithelial cells were diluted in DMEM medium supplemented and in BEBM medium with 10% FBS (Thermo Fisher Scientific, Inc.). Cells suspension, at a concentration of 1 × 10^4^ cells/mL, was inoculated into 96-well culture plate, 180 µL per well, and cultured for 24 h at 37 °C in a fully humidified atmosphere of 5% CO_2_ in air. After the cells adhered to the wells, LLF were added at a volume of 20 µL per well but at different concentrations (0–500 µg/mL). After 48 h of LLF treatment, 20 µL MTT reagent (5 mg/mL) were added to each well follower by further incubation for 4 h. At the end of culture, the supernatant was aspirated and discarded, DMSO was added to at a volume of 150 µL per well and the cells were cultured for 30 min. The optical density (OD) value of each well was measured by enzyme labeling instrument at 570 nm wavelength, and the inhibition rate of cell proliferation was calculated [16] according to the following formula: Inhibition rate (%) = [(blank well OD value − sample well OD value)/blank well OD value] × 100.

### Live cell count

Human lung cancer A549 cells were diluted in DMEM medium supplemented and in BEBM medium with 10% FBS (Thermo Fisher Scientific, Inc.). Cells suspension, at a concentration of 1 × 10^4^ cells/mL, was inoculated into 96-well culture plate, 180 µL per well, and cultured for 24 h at 37 °C in a fully humidified atmosphere of 5% CO_2_ in air. After the cells adhered to the wells, LLF were added at a volume of 20 µL per well but at different concentrations (125, 250 and 500 µg/mL). At 12, 24, 36 and 48 h after LLF treatment, the medium was discarded and 200 µL trypsin was added in each well. Then the cells were made into single cell suspension and counted under the microscope.

### Detection of apoptosis by flow cytometry

A549 cells in the logarithmic growth phase were treated with LLF at 25, 50 and 100 µg/mL for 48 h. 1 × 10^6^ cells were collected, washed with PBS, precooled ethanol was slowly added into 3 mL 70%, and fixed overnight at 4 °C, the cells were suspended in Annexin V-FITC binding solution (Thermo Fisher Scientific, Inc.) was added, gently mixed, and incubated at 4ºC in the dark for 15 min. FACScalibur flow cytometer (BD Biosciences, Franklin Lakes, NJ, USA) was used to analyze the distribution of cell cycle [17], and Mod Fit software was used for analysis.

### Detection of ROS by flow cytometry

Cells in the same LLF concentrations as above group were collected after LLF treatment, and 10 µmol/L ROS-detecting dye (DCFH-DA) was added to cells before the ROS-inducing stimulus, washed three times with PBS, then incubated at 37 °C for 20 min. Following incubation, the cells were washed three times with PBS, resuspended in RPMI 1640 medium, and the fluorescence intensity of each test sample was detected by flow cytometry (BD Biosciences), with mean fluorescence intensity (MFI) representing ROS content [18].

### Detection of SOD activity and MDA content

Cells in the same LLF concentrations as above group were collected after LLF treatment and washed three times with PBS. After washing, the cells were lysed with RIPA buffer to extract the total protein separately in each group. Ultraviolet spectrophotometry was used to determine protein concentration. WST-8 was used to detect the activity of SOD and thiobarbituric acid was used to detect the content of MDA. Detection was carried out according to the methods and operation requirements of each kit (Solarbio Life Sciences, Beijing, China).

### Quantitative real time PCR (qRT-PCR) determination

The culture medium of A549 cells was discarded, the adherent cells were washed with PBS, and then the total RNA was extracted by RNAzol (Thermo Fisher Scientific, Inc.), and diluted to 1 µg/µL. Total RNA solution (5 µL) was used for reverse transcription according to the kit’s instructions to obtain cDNA template. cDNA template (2 µL), 10 µL of SYBR Green PCR master mix (Thermo Fisher Scientific, Inc.), and 1 µL each of upstream and downstream primers (Table 3) were mixed and reacted at 95 °C for 60 s, then 40 cycles of 95 °C for 15 s; 55 °C for 30 s; 72 °C for 35 s. GAPDH was used as an internal standard. 2^−ΔΔCT^ method was used to calculate the relative expression of genes [19].

**Table 3:** Sequences of primers used in the qPCR assay

Gene Name	Sequence
Cu/Zn-SOD	Forward: 5′GGTGGGCCAAAGGATGAAGAG-3′
	Reverse: 5′CCACAAGCCAAACGACTTCC-3′
CAT	Forward: 5′-TGGAGCTGGTAACCCAGTAGG-3′
	Reverse: 5′CCTTTGCCTTGGAGTATTTGGTA-3′
Nrf2	Forward: 5′-CTTTTGCGCAGACATTCCC-3′
	Reverse: 5′-GACTGGGCTCTCGATGTGAC-3′
NQO1	Forward: 5′-GGTTTGAGCGAGTGTTCATAGG-3′
	Reverse: 5′-GCAGAGAGTACATGGAGCCAC-3′
HO-1	Forward: 5′-CAGTGCCAACCAAGTTCAAGC-3′
	Reverse: 5′-GTTGAGCAGGAACGCAGTCTT-3′
p38 MAPK	Forward: 5′-CTACCCGCAGGAGCTGAACAA-3′
	Reverse: 5′-AATCATGGACTGAAATGGTCTCCAG-3′
Caspase-3	Forward: 5′-CATGGAAGCGAATCAATGGACT-3′
	Reverse: 5′-CTGTACCAGACCGAGATGTCA-3′
Caspase-9	Forward: 5′-CTCAGACCAGAGATTCGCAAAC-3′
	Reverse: 5′-GCATTTCCCCTCAAACTCTCAA-3′
Bax	Forward: 5′-CCAAGGTGCCGGAACTGA-3′
	Reverse: 5′-CCCGGAGGAAGTCCAATGT-3′
Bcl-2	Forward: 5′-TGCGGCCTCTGTTTGATTTC-3′
	Reverse: 5′-GGGCCAAACTGAGCAGAGTCT-3′
GAPDH	Forward: 5′-TCAAGAAGGTGGTGAAGCAGG-3′
	Reverse: 5′-AGCGTCAAAGGTGGAGGAGTG-3′

### Western blot detection

The culture medium of cancer cells was discarded, the adherent cells were washed with PBS, and then the total proteins were extracted using 1 mL of RIPA buffer with 10 µL PMSF. Extracted proteins from each group were diluted to 50 µg/mL, and then further diluted at a rate of 4:1 with sample buffer. The mix was heated at 100ºC for 5 min. Acrylamide, resolving buffer, starting buffer, deionized water, 10% APS, and TEMED were mixed in proportion to make SDS-PAGE separation gel (Thermo Fisher Scientific, Inc.), which was poured into a rubber plate. Pre-stained Protein Ladder and samples were placed into the sample wells and the SDS-PAGE gel was subjected to 50 min of vertical electrophoresis. The PVDF membrane was activated with methanol for 1 min, and was then blocked with 5% skimmed milk in TBST solution (Thermo Fisher Scientific, Inc.) for 1 h. After blocking, the PVDF membrane was washed with TBST, incubated with primary antibody (Cu/Zn-SOD, ab51254, 1/50,000 dilution; CAT, ab209211, 1/2000 dilution; Nrf2, ab62352, 1/1000 dilution; NQO1, ab80588, 1/10,000 dilution; HO-1, ab189491, 1/2000 dilution; caspase-3, ab13847, 1/500 dilution; caspase-9, ab32539, 1/1000 dilution; cleaved caspase-3, ab32042, 1/500 dilution; cleaved caspase-9, ab2324, 1/500 dilution; Abcam, Cambridge, UK; p-p38 MAPK, 44-684G, 1/1000 dilution, Thermo Fisher Scientific, Inc.) at 25 °C for 2 h, and then with secondary antibody at 25 °C for 1 h. Finally, protein bands on the PVDF membrane were detected using Supersignal West Pico plus inside iBright FL1000 (Thermo Fisher Scientific, Inc.) [20].

### Statistical analysis

All experiments were performed three times in parallel, and the average value was used. The SAS 9.1 statistical software was used for data analysis. One-way ANOVA was used to compare between groups with the significance level set at *P* < 0.05.

## Results

### Composition analysis of lotus leaves flavonoids

**Fig. 1:**
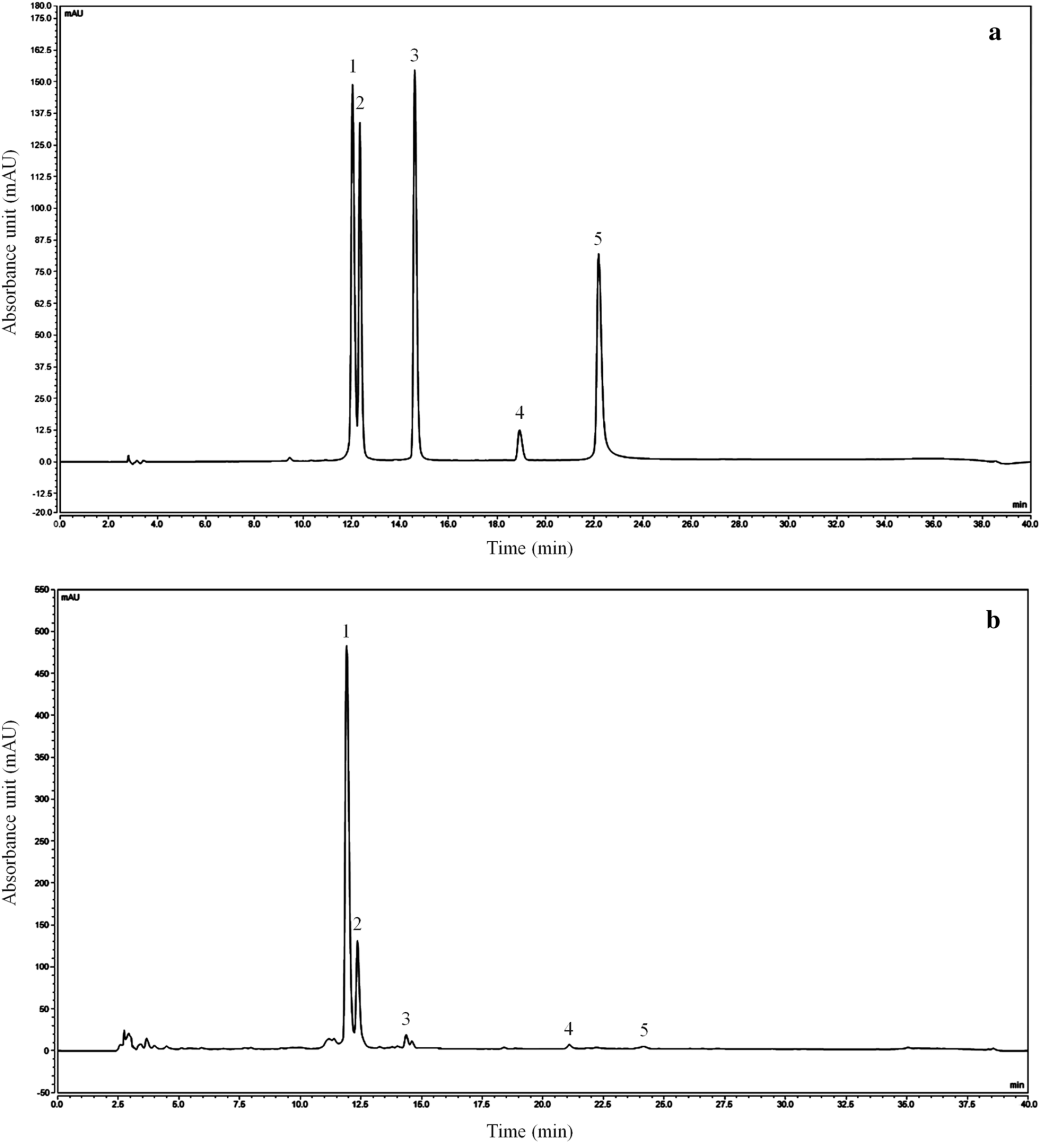
Flavonoids extract constituents of lotus leaves. **a** Standard chromatograms; **b** Flavonoids of lotus leaves chromatograms. *1* kaempferitrin; *2* hyperoside; *3* astragalin; *4* phloridzin; *5* quercetin, (n = 3)

Through analysis of the liquid phase, we know that the flavones of lotus leaves include mainly kaempferitrin, hyperoside, astragalin, phloridzin, and quercetin, among which kaempferoside and hyperoside are the most abundant (Fig. 1). The content purities of kaempferitrin, hyperoside, astragalin, phloridzin, and quercetin were 378.93 mg/g, 155.57 mg/g, 47.62 mg/g, 1.27 mg/g and 0.48 mg/g, respectively.

### Effect of lotus leaves flavonoids on cell viability

As can be seen from Table 4, LLF had no inhibitory effect on the viability of normal lung BEAS-2B cells. However, under these same concentrations, viability of A549 and H446 lung cancer cells was inhibited by LLF, but LLF had little effect on the cells after 24 h. The rate of viability was proportional to LLF concentration. At concentrations of 125, 250 and 500 µg/mL, LLF inhibited A549 cells’ viability by 21.52%, 55.91% and 83.33% (Table 5), and also inhibited A549 cells’ viability by 20.77%, 49.29% and 81.26% (Table 6). The IC50 was calculated by graphpad prism software [Log(inhibitor) vs. response-Variable slope(four parameters)]. And the IC50 of LLF in A549 and H446 lung cancer cells were 282.1 ± 9.63 and 292.9 ± 11.22 µg/mL, respectively. Based on these observations, these three concentrations were used in subsequent experiments.

**Table 4:** Viability of BEAS-2B lung bronchus epithelial cells by different concentration of lotus leaves flavonoids (LLF) as evaluated by an MTT assay (n = 6)

Concentration (µg/mL)	OD_570_	Viability inhibition rate (%)
0 (Control)	0.486 ± 0.006	/
125	0.473 ± 0.003^*^	2.66 ± 1.45
250	0.465 ± 0.003^*^	4.38 ± 1.49^#^
500	0.445 ± 0.003^*^	8.46 ± 1.26^##^

Values presented are the mean ± standard deviation. Compared with the control group, the difference was significant (^*^p < 0.05; ^**^p < 0.01); and Compared with the 125 µg/mL LLF group, the difference was significant (^#^*p* < 0.05; ^##^ *p* < 0.01)

**Table 5:** Viability of A549 lung cancer cells by different concentration of lotus leaves flavonoids (LLF) as evaluated by an MTT assay (n = 6)

Concentration (µg/mL)	OD_570_	Viability inhibition rate (%)
0 (Control)	0.474 ± 0.006	/
125	0.372 ± 0.015^*^	21.52 ± 2.56
250	0.209 ± 0.008^*^	55.91 ± 2.63^#^
500	0.079 ± 0.005^**^	83.33 ± 0.69^##^

Values presented are the mean ± standard deviation. Compared with the control group, the difference was significant (^*^p < 0.05; ^**^p < 0.01); and Compared with the 125 µg/mL LLF group, the difference was significant (^#^*p* < 0.05; ^##^*p* < 0.01)

**Table 6:** Viability of H446 lung cancer cells by different concentration of lotus leaves flavonoids (LLF) as evaluated by an MTT assay (n = 6)

Concentration (µg/mL)	OD_570_	Viability inhibition rate (%)
0 (Control)	0.491 ± 0.011	/
125	0.389 ± 0.015^*^	20.77 ± 2.30
250	0.249 ± 0.021^*^	49.29 ± 2.18^#^
500	0.092 ± 0.011^**^	81.26 ± 1.87^##^

Values presented are the mean ± standard deviation. Compared with the control group, the difference was significant (^*^p < 0.05; ^**^p < 0.01); and Compared with the 125 µg/mL LLF group, the difference was significant (^#^*p* < 0.05; ^##^*p* < 0.01)

### Effect of lotus leaves flavonoids on cells proliferation

**Fig. 2:**
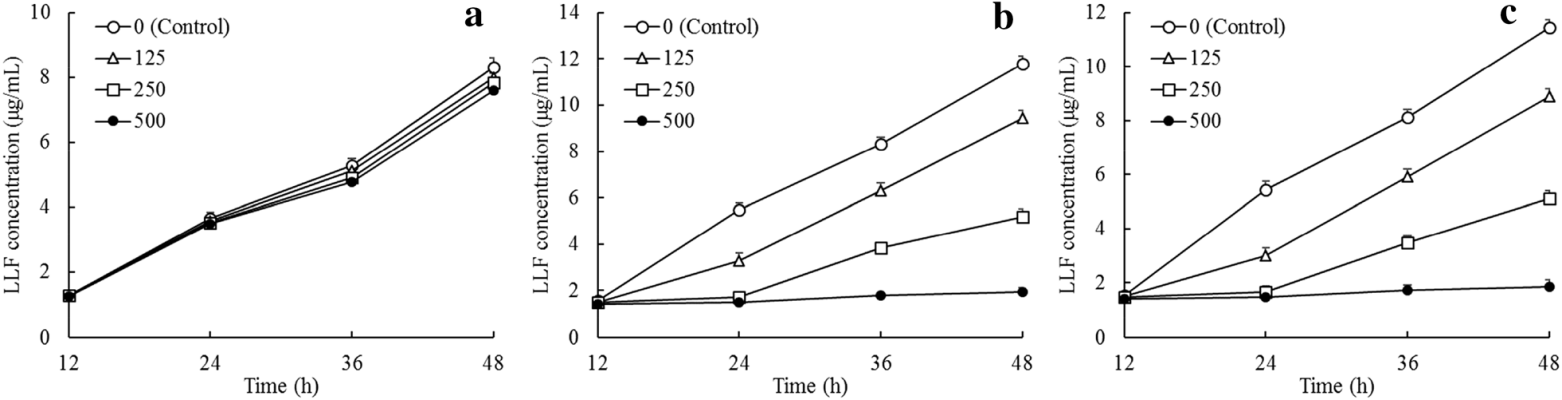
Growth inhibition of BEAS-2B lung bronchus epithelial cells (**a**), A549 lung cancer cells (**b**) and H446 lung cancer cells (**c**) by different concentration of lotus leaves flavonoids (LLF) (n = 6)

As can be seen from Fig. 2, after treatment with LLF for 12, 24, 36 and 48 h, at concentrations of 125, 250 and 500 µg/mL, LLF could raise the inhibitory rate in A549 and H446 cancer cells, and the higher concentration showed the stronger inhibitory effects.

### Effect of lotus leaves flavonoids on A549 cells cycle progression

**Fig. 3:**
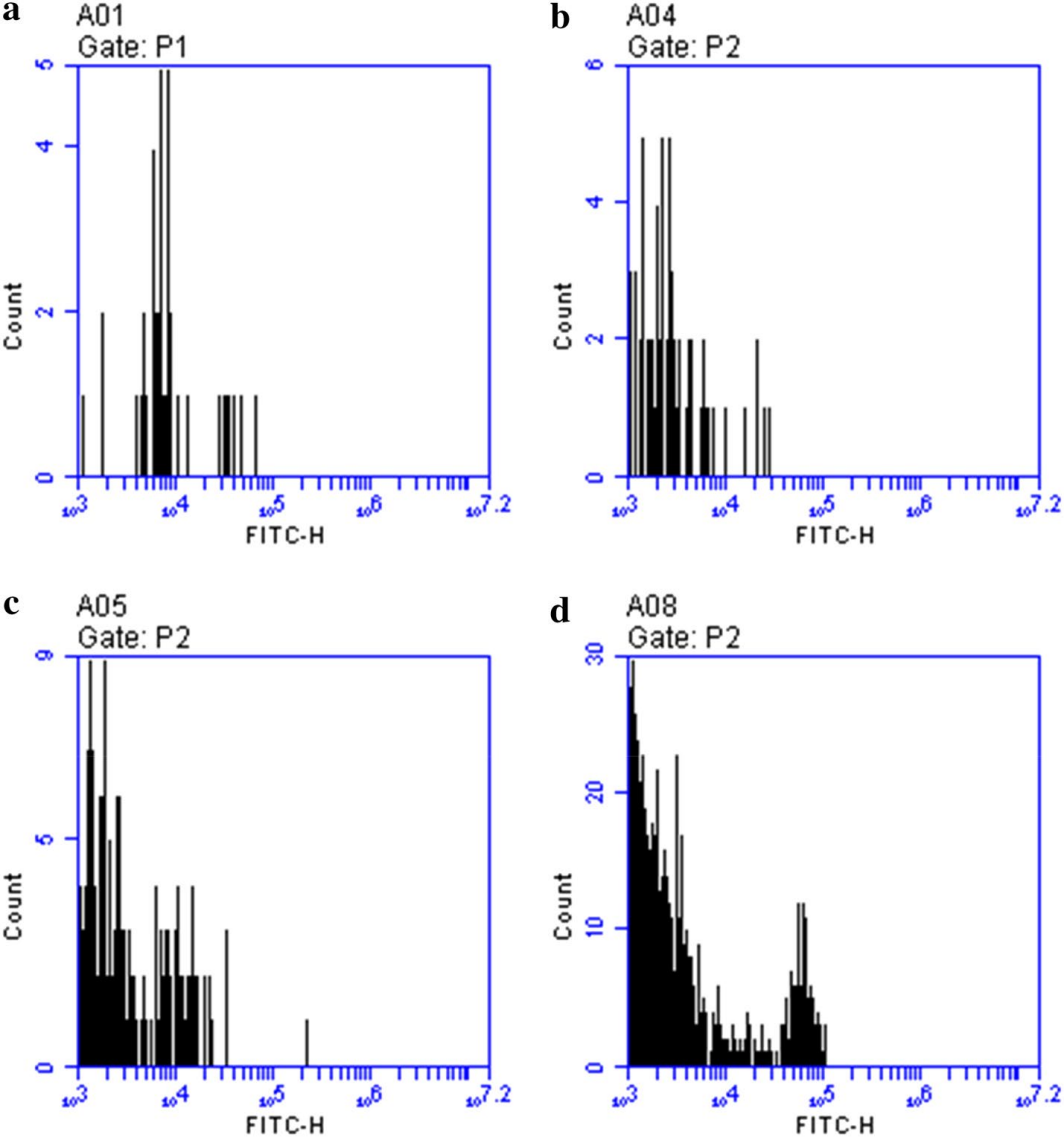
Effect of lotus leaves flavonoids (LLF) on the apoptosis marker Annexin-V over A549 lung cancer cells. A549 cells were exposed to LLF at different concentrations and the fluorescence intensity after Annexin-V stain was measured by flow cytometry. **a** Control: untreated A549 cells; **b** A549 cells treated with LLF at 125 µg/mL for 48 h; **c** A549 cells treated with LLF at 250 µg/mL for 48 h; **d** A549 cells treated with LLF at 500 µg/mL for 48 h. Frequency histogram represents the median fluorescence intensity (MFI) against the cell count. The increase in the MFI represents an increase in the number of the cells positive for the apoptosis mark Annexin-V (n = 6)

As shown in Fig. 3, phase S of A549 cells was induced by 500 µg/mL LLF, and, compared A549 cells control group, the proportions of cells in fragments and cells at the S phase were higher. Most of the cells remained in the S phase and could not progress to the G2 phase. Similarly, A549 cells treated with 125 µg/mL and 250 µg/mL LLF remained in the S phase, and apoptosis cells and cell fragments were higher than the control group, but the change was smaller than that in the 500 µg/mL LLF group. With the increase of LLF concentration, the number of dead cells increased.

### Effect of lotus leaves flavonoids on ROS content in A549 cells

**Fig. 4:**
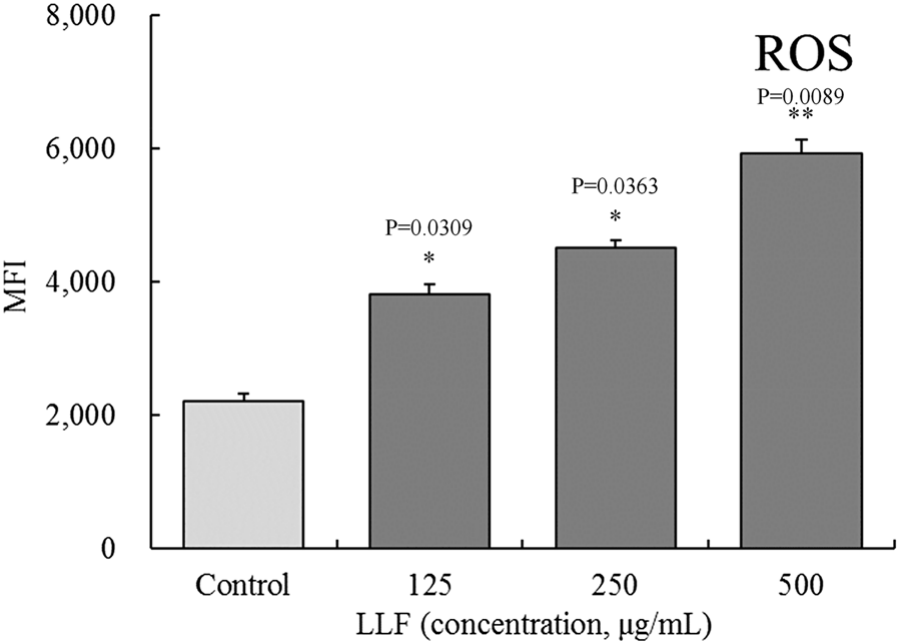
Effect of lotus leaves flavonoids (LLF) on ROS content in A549 lung cancer cell. Control: untreated A549 cells. Compared with the control group, the difference was significant (^*^p < 0.05; ^**^p < 0.01). Control: untreated A549 cells. Cells were treated with LLF at 125, 250 and 500 µg/mL for 48 h (n = 6)

As the results of flow cytometry shown in Fig. 4 indicate, the mean fluorescence intensities (MFIs) in the 125, 250 and 500 µg/mL LLF groups were all significantly higher than in the control group (*P* < 0.05).

### Effect of lotus leaves flavonoids on SOD activity and MDA content in A549 cells

**Fig. 5:**
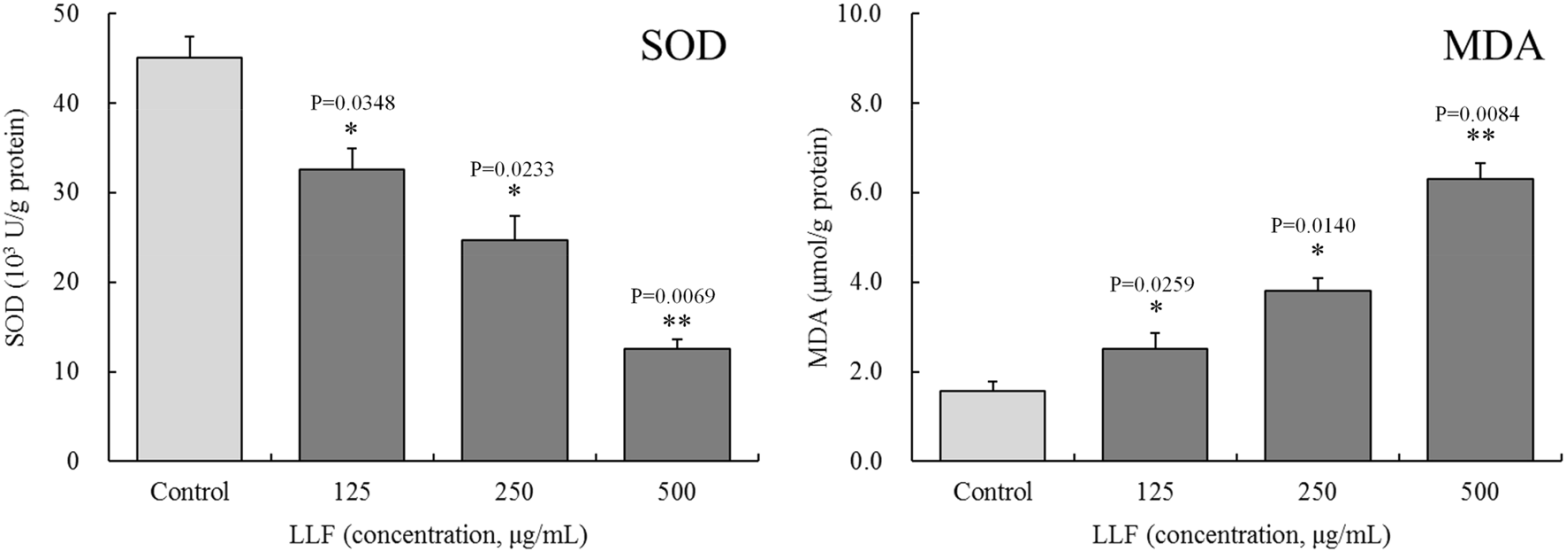
Effect of lotus leaves flavonoids (LLF) on SOD activity and MDA content in A549 lung cancer cell. Control: untreated A549 cells. A549 cells were treated with LLF at 125, 250 and 500 µg/mL for 48 h. Compared with the control group, the difference was significant (^*^p < 0.05; ^**^p < 0.01). Control: untreated A549 cells (n = 6)

As can be noted in Fig. 5, the results of WST-8 and thiobarbituric acid evaluations show that, compared with the control group, SOD activity following treatment with 125, 250, and 500 µg/mL LLF has decreased significantly in all three treatment groups (*P* < 0.05), whereas MDA contents increased significantly (*P* < 0.05). There was significant difference in SOD activity and MDA content between the treatment groups (*P* < 0.05).

### Cu/Zn-SOD and CAT mRNA and protein expression in A549 cells

**Fig. 6:**
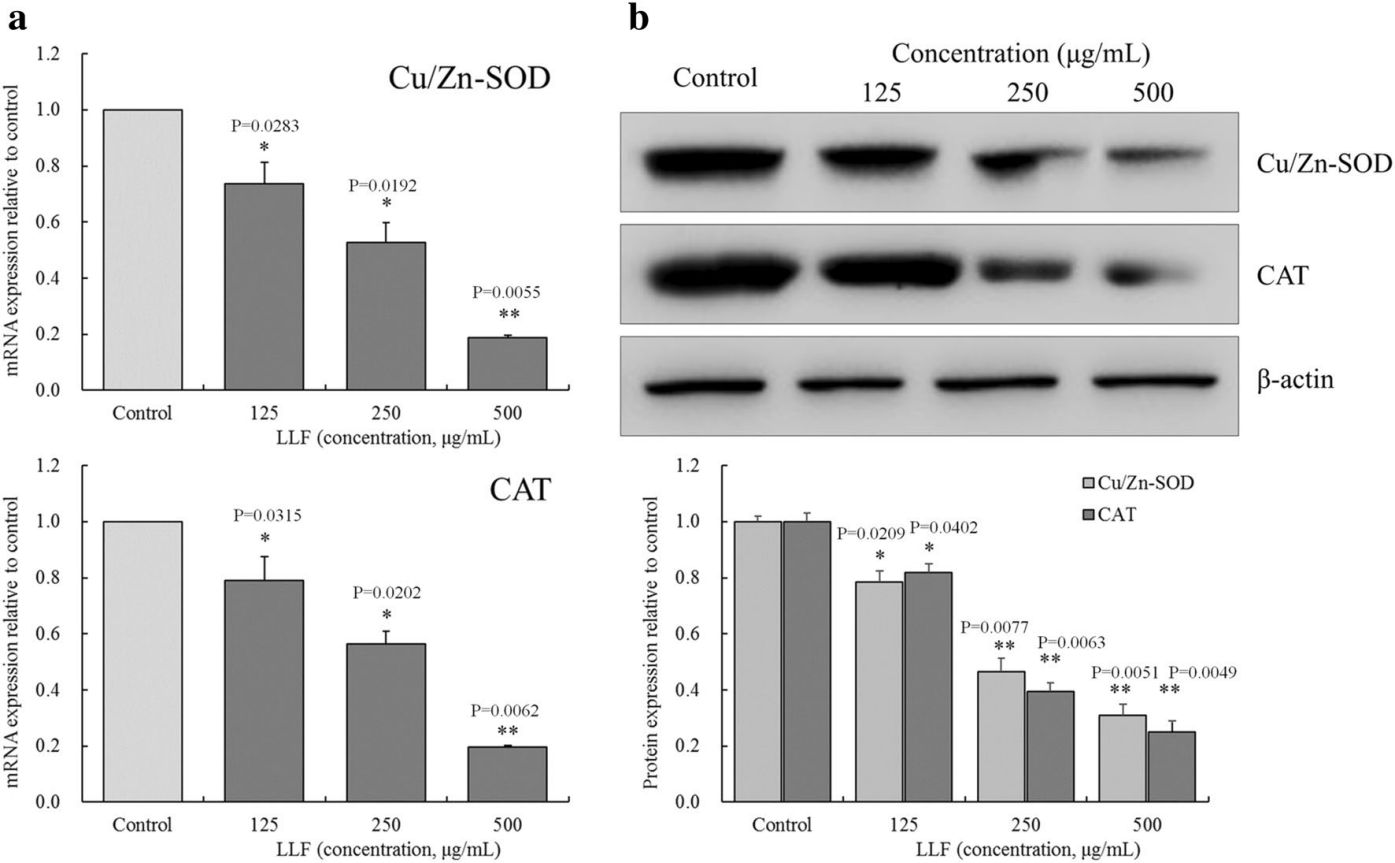
mRNA (**a**) and protein (**b**) expression of Cu/Zn-SOD and CAT on the lotus leaves flavonoids (LLF) treated A549 lung cancer cells. A549 cells were treated with LLF at 125, 250 and 500 µg/mL for 48 h. Values presented are the mean ± standard deviation. Compared with the control group, the difference was significant (^*^p < 0.05; ^**^p < 0.01). Control: untreated A549 cells (n = 3)

As shown in Fig. 6, the mRNA and protein expression of Cu/Zn-SOD and CAT were the highest in the control group. This indicate that LLF caused a significant (*P* < 0.05) decrease in the expression of Cu/Zn SOD and CAT in A549 cells, and the ability of LLF to regulate the expression of Cu/Zn-SOD and CAT was positively correlated with LLF concentration.

### Nrf2, NQO1and HO-1 mRNA and protein expression in A549 cells

**Fig. 7:**
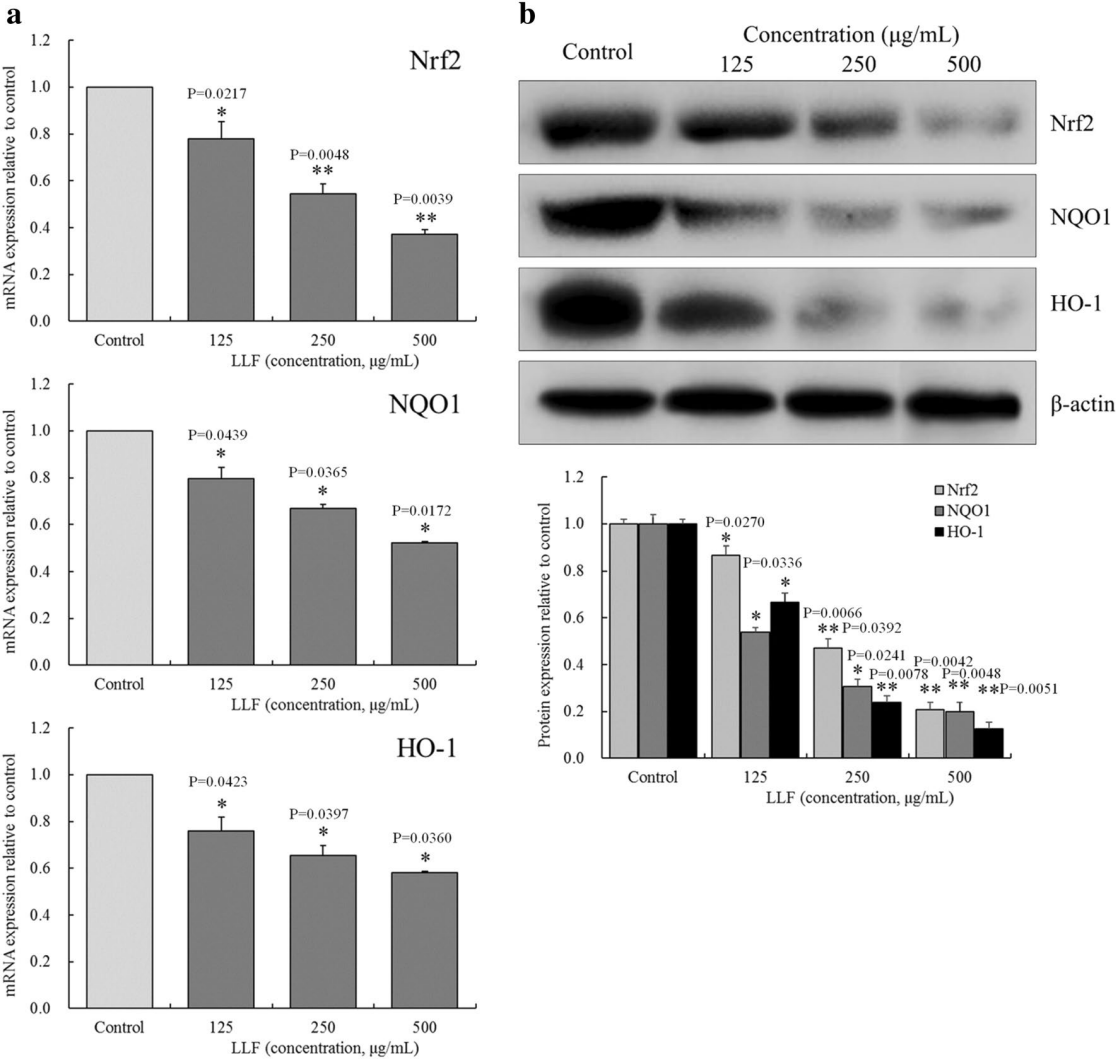
mRNA (**a**) and protein (**b**) expression of Nrf2, NQO1and HO-1 on the lotus leaves flavonoids (LLF) treated A549 lung cancer cells. A549 cells were treated with LLF at 125, 250 and 500 µg/mL for 48 h. Values presented are the mean ± standard deviation. Compared with the control group, the difference was significant (^*^p < 0.05; ^**^p < 0.01). Control: untreated A549 cells (n = 3)

As shown in Fig. 7, The mRNA and protein expression of Nrf2, NQO1, and HO-1 were the highest in the control group. As the concentration of LLF increased, their expression decreased.

### p38 MAPK and caspases mRNA and protein expression in A549 cells

**Fig. 8:**
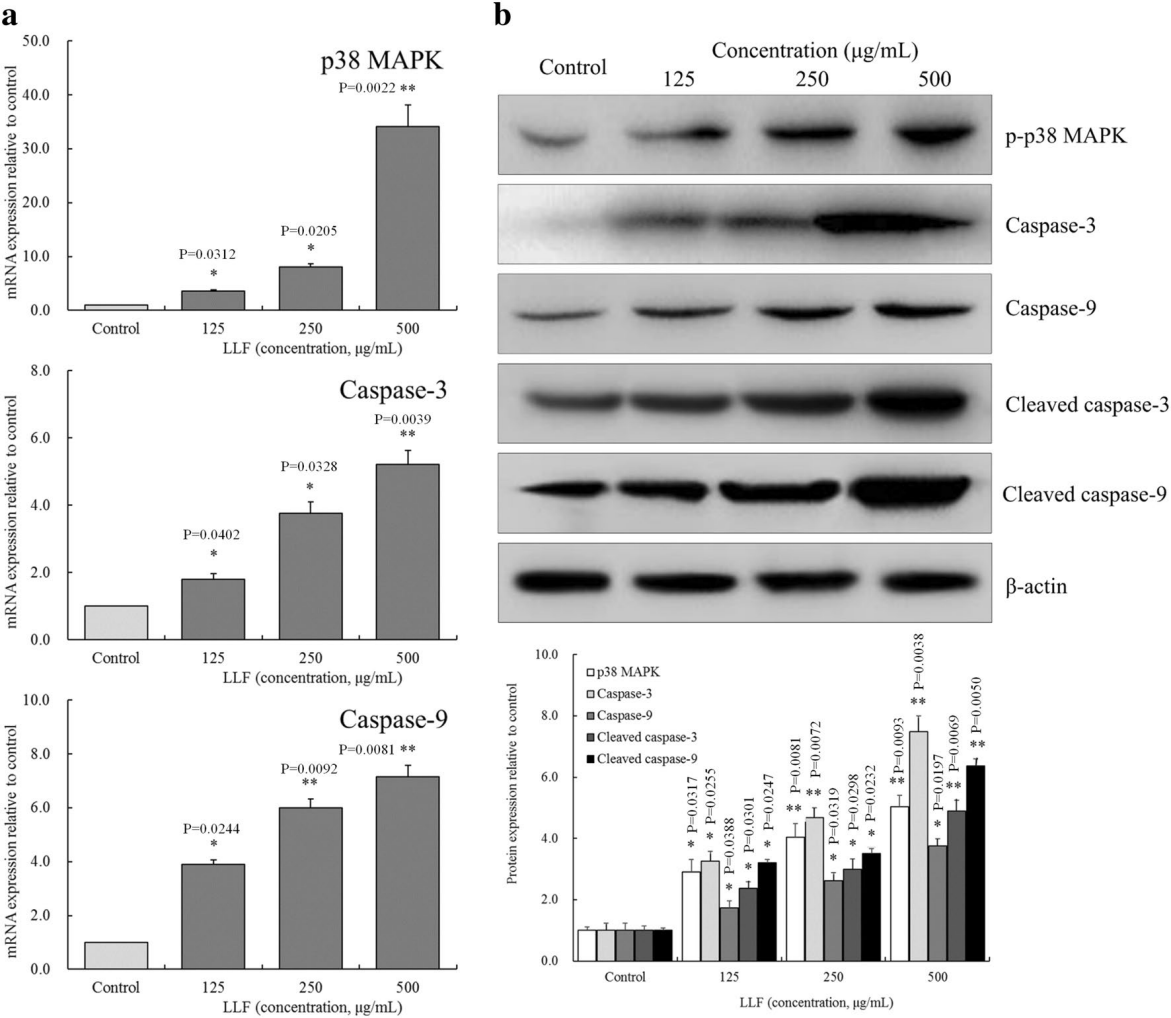
mRNA (**a**) of p38 MAPK, caspase-3, caspase-9 and protein (**b**) expression of p-p38 MAPK, caspase-3, caspase-9, cleaved caspase-3, cleaved caspase-9 on the lotus leaves flavonoids (LLF) treated A549 lung cancer cells. A549 cells were treated with LLF at 125, 250 and 500 µg/mL for 48 h. Values presented are the mean ± standard deviation. Compared with the control group, the difference was significant (^*^p < 0.05; ^**^p < 0.01). Control: untreated A549 cells (n = 3)

As shown in Fig. 8, the mRNA expression of p38 MAPK, capase-3, caspase-9 were the lowest in the control group, as were p-p38 MAPK, caspase-3, caspase-9, cleaved caspase-3, cleaved caspase-9 proteins. LLF could significantly (*P* < 0.05) upregulate these expressions in A549 cells.

### Bax and Bcl-2 mRNA and protein expression in A549 cells

**Fig. 9:**
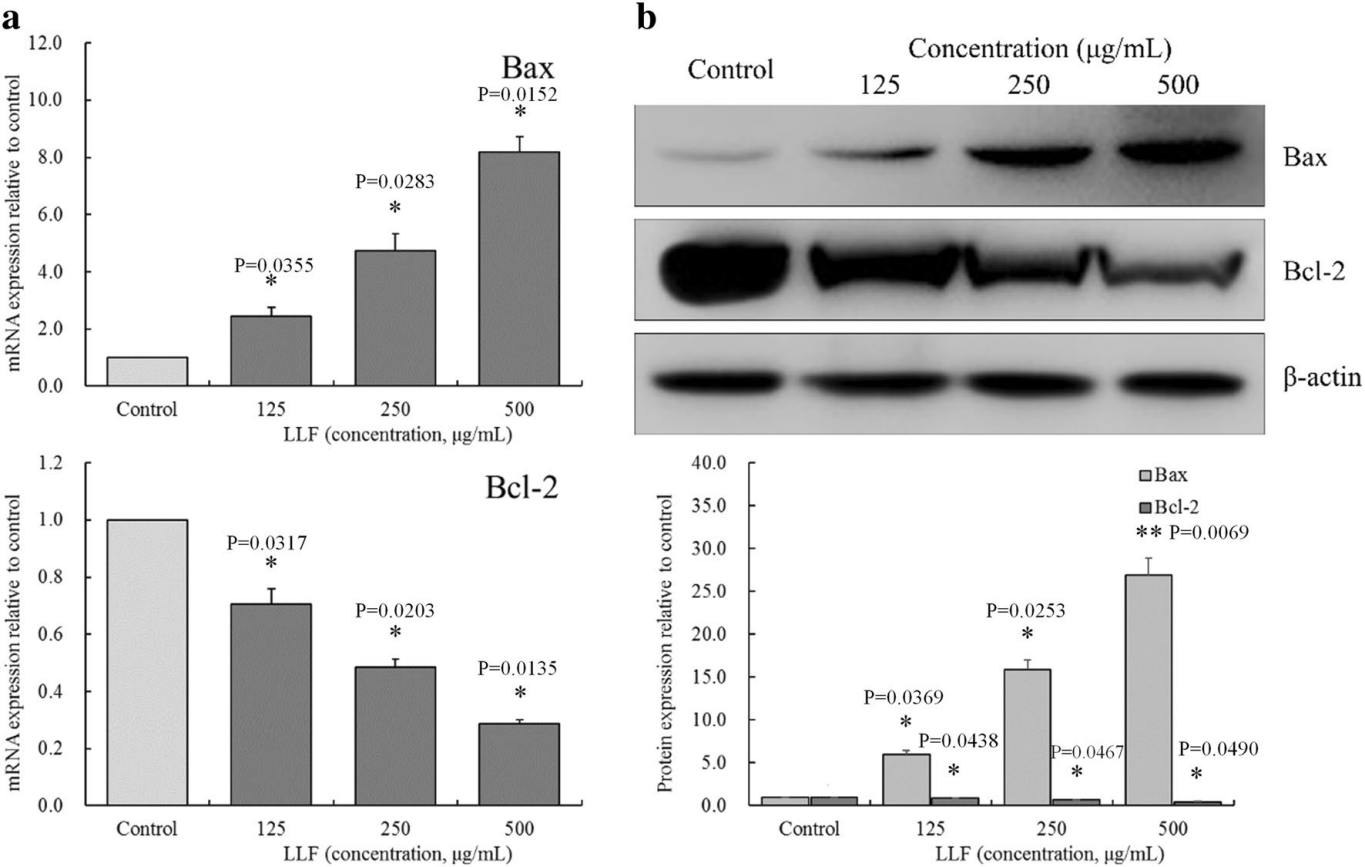
mRNA (**a**) and protein (**b**) expression of Bax and Bcl-2 on the lotus leaves flavonoids (LLF) treated A549 lung cancer cells. A549 cells were treated with LLF at 125, 250 and 500 µg/mL for 48 h. Values presented are the mean ± standard deviation. Compared with the control group, the difference was significant (^*^p < 0.05; ^**^p < 0.01). Control: untreated A549 cells (n = 3)

As shown in Fig. 9, expression of Bax mRNA and protein were the lowest, while the expression of Bcl-2 mRNA and protein were the highest in the control group. After treating A549 cells with LLF, the expression of Bcl-2 decreased and expression of Bax increased in a dose-dependent manner.

### Effects of ROS scavenger NAC (*N*-acetyl-l-cysteine) and p38 MAPK inhibitor on p-p38 MAPK protein in LLF treated A549 cells

**Fig. 10:**
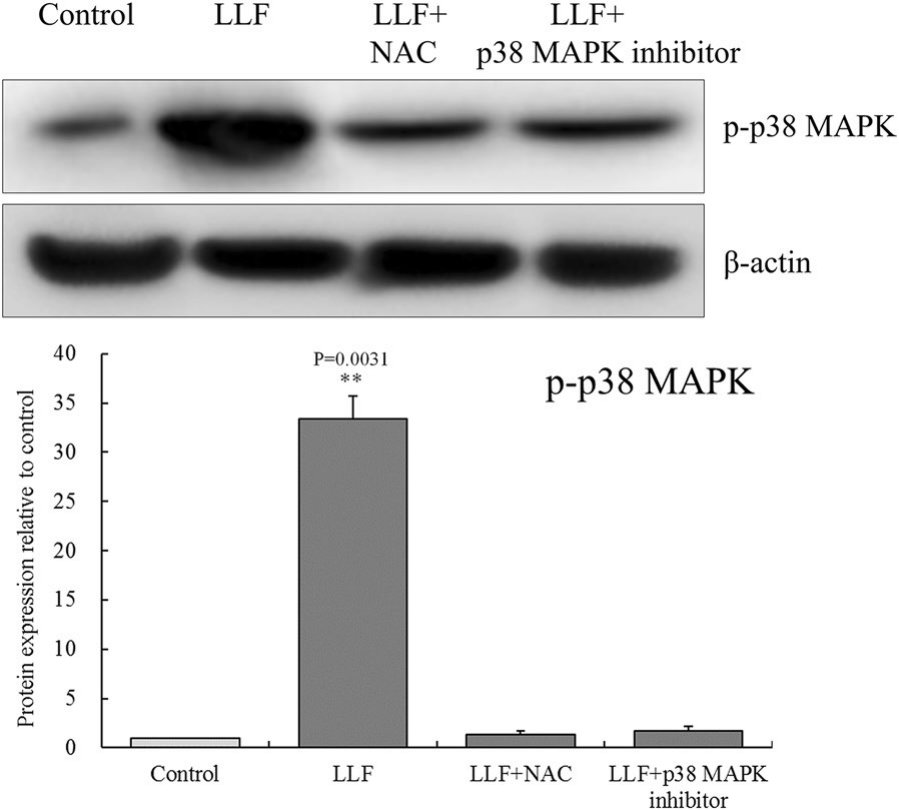
Protein expression of p-p38 MAPK on the lotus leaves flavonoids (LLF), ROS scavenger NAC (*N*-acetyl-l-cysteine) and sb203580 p38 MAPK inhibitor treated A549 lung cancer cells. A549 cells were treated with LLF at 500 µg/mL, LLF (500 µg/mL) + NAC (800 µg/mL) and LLF (500 µg/mL) + p38 MAPK inhibitor (4 µg/mL) for 48 h. Values presented are the mean ± standard deviation. Compared with the control group, the difference was significant (^**^p < 0.01). Control: untreated A549 cells (n = 3)

After treated with NAC (800 µg/mL) or sb203580 p38 MAPK inhibitor (4 µg/mL) for 1 h, LLF (500 µg/mL) was added to continue treatment. As shown in Fig. 10, after A549 cells were treated with LLF alone, LLF + NAC and LLF + p38 MAPK inhibitor, we found that the p-p38 MAPK protein of A549 cells was significantly (*p* < 0.01) increased under the action of LLF alone, while NAC and p38 MAPK inhibitors could significantly (*p* < 0.01) inhibit the increase of p-p38 MAPK protein induced by LLF.

## Discussion

Cell signal transduction is a process in which extracellular factors bind with membrane and/or nuclear receptors to initiate a series of biochemical reactions and protein-protein interactions in the cell, leading to expression of genes required for targeted physiological reactions [21]. Apoptosis, on the other hand, refers to the spontaneous and orderly genes-controlled cell death that helps maintain stability in the internal environment. Apoptosis is different from necrosis [22]. In this study, LLF could regulate the gene expression of A549 cancer cells, which may play a role in regulating the death or apoptosis pathway of A549 cancer cells.

The resulting elevated concentration of ROS causes apoptosis [23]. ROS released can cause cell damage and even death. ROS can also promote the activation of caspase-9, thus promoting the apoptosis of cancer cells [24]. Meanwhile ROS can activate caspase 2 and 9 and act on the downstream target caspase zymogens (caspases 3, 6, and 7) and other targets. The activated caspase 2 and 3 act as effectors on different targets in the cells, and cause apoptosis [25]. In this study, LLF can also promote the production of ROS in A549 cancer cells, thus affecting the proliferation of A549 cancer cells, including the role of promoting apoptosis by affecting caspases. In further studies, the mechanism of LLF regulating caspases through ROS needs to be further studied.

Biological active substances can induce apoptosis of cancer cells by causing the accumulation of ROS [26]. Disbalance between the pro-oxidative state and the antioxidants defenses of the cell can raise ROS level, this case can oxidize the phospholipids in the cellular membrane to form stable malondialdehyde (MDA), a mutagenic product of lipid peroxidation. This leads to cellular damage and apoptosis [27]. Superoxide dismutase (SOD) is one of the main enzymes that remove ROS. Human cells are rich in SOD1 (Cu/Zn-SOD), so detection of SOD1 can significantly reflect the activity of SOD in the cells [28]. Increased SOD content in A549 cells can reduce apoptosis that was caused by ROS accumulation. Therefore, reducing SOD activity in cancer cells can promote their apoptosis [29]. Catalase can also promote the decomposition of hydrogen peroxide in cancer cells, so that they will not continue to produce toxic free radicals and thus avoid apoptosis [30]. In this study, LLF were shown to increase ROS in A549 cells, and promote their apoptosis by raising MDA content and reducing SOD and CAT activities.

Nrf2 is a key molecule expressed during cellular oxidative stress. By binding with anti-oxidative response elements genes, Nrf2 regulates the transcription and expression of a variety of antioxidant enzymes, such as NQO1 and HO-1 [31]. It has been confirmed that Nrf2 knockout can down regulate the expression of NQO1 and HO-1 proteins in A549 cells, and increase ROS level in these cells [32]. Silencing NQO1 gene by shRNA can also increase ROS level in A549 cells, as can the silencing of HO-1 gene [33]. The response of Nrf2 mediated antioxidant system is related to its intracellular regulation. At low dose of Nrf2 activator, most of the effectors of Nrf2/ARE pathway can provide cell protection. In the presence of excessive ROS, continuous activation of Nrf2 leads to its accumulation in the nucleus and subsequent binding to the Kruppel like factor 9 (KLF9) promoter. Up regulation of KLF9 transcription is a new intracellular ROS regulator, which may lead to increased ROS levels and subsequent cell death [34]. In this study, LLF could regulate the expression of Nrf2, NQO1, and HO-1 to maintain a high level of ROS and, in this manner, induce apoptosis in A549 cells.

According to the role of caspases in apoptosis and the length of their N-terminal, they can be divided into two categories: one is the initiating type and the other is the effector caspase. The former includes caspase 2, 8, 9, and 10, which have long N-terminal and can initiate apoptosis and regulate its downstream effector caspases. The latter includes caspase 3, 6, and 7, which have short N-terminal and are the final effectors of apoptosis [35]. There are two mechanisms of caspase release and action in mitochondria. After caspase is activated by a death signal, it can activate a second messenger such as Ca^2+^, Bcl-2, ceramide, active oxygen, etc., which acts on the mitochondria. After procaspase 2, 3, or 9 is activated by the release of such factors from the mitochondria, it can activate the downstream caspases 3, 6, and 7. Activated caspases 3, 6, and 7 react on p38 kinase, induce further release of MPT, and caspases 2, 3, and 9, that together realize a cascade amplification of death signal, and accelerate apoptosis. Similarly, cytochrome c can also be released to the cytoplasm, a process promoted by MPT. When cytochrome c binds to pro-caspase 9, it activates it, and consequentially activates caspase 3, and the rest of the caspase cascade reaction that leads to apoptosis [36]. LLF could enhance the expression of caspase-3 and caspase-9 in cancer cells, playing an inhibiting role on these cells. In addition, cleaved caspases can display endogenous caspase-3 protein when caspases are cleaved and activated [34]. In this study, LLF can regulate the protein expression of cleaved caspase-3 and cleaved caspase-9, thus affecting caspase-3 and caspase-9, and promoting cell apoptosis.

The Bcl-2 gene is a kind of cancer gene, which can inhibit apoptosis. At present, the Bcl-2 protein family can be divided into two categories, according to their functions. Some genes, similar to Bcl-2, can inhibit apoptosis, whereas others, such as Bax, can promote it [37]. In this study, LLF was shown to promote cancer cells’ apoptosis by inhibiting bcl-2 expression and enhancing Bax expression.

ROS can continuously activate p38 MAPK by activating MAPK kinase and inhibiting MAPK phosphatase. p38 MAPK plays an important role in ROS-induced apoptosis in lung cancer cells. In A549 cells, ROS can upregulate the expression of Bax and Bcl-2 by activating p38 MAPK, which can increase the level of cytochrome c in the cytoplasm and trigger the caspase cascade reaction that leads to apoptosis [38]. The active substance can activate p38 MAPK and caspase-3 in A549 cells, and induce apoptosis by activating the ROS/p38 MAPK pathway [39]. In this study, ROS scavenger NAC and p38 MAPK inhibitor could reduce the expression of p-p38 MAPK in LLF treated A549 cells, LLF played a similar role by activating ROS/p38 MAPK pathway and promoting apoptosis of A549 cells, an effect that was positively correlated with the concentration of LLF.

**Fig. 11:**
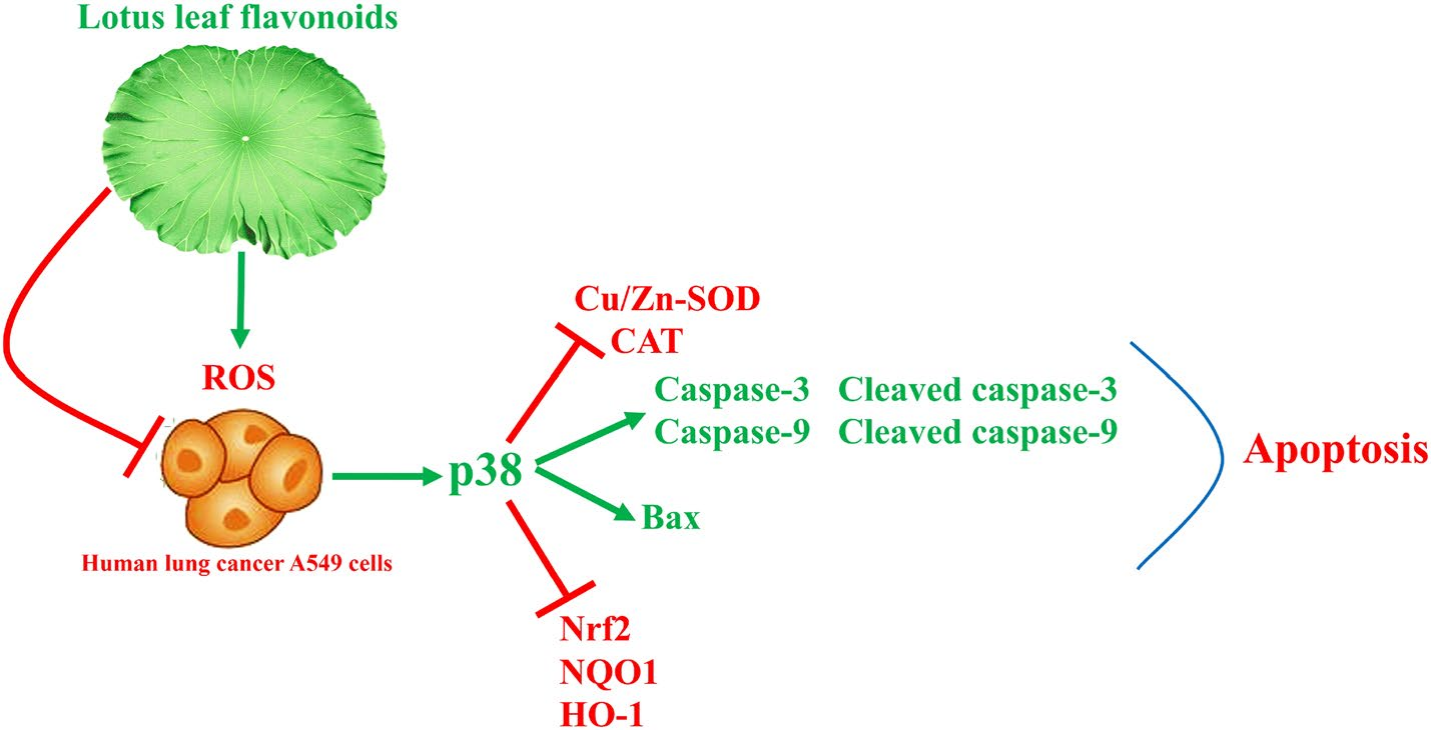
Mechanism of action of lotus leaves flavonoids (LLF) on A549 lung cancer cells

Kaempferitrin is a natural flavonoid glycoside. Kaempferitrin was shown to have analgesic, anti-inflammatory, anti-diabetic, anti-tumorigenic, and cancer therapeutic effects. Furthermore, it can cause cell cycle arrest at the G1 phase, and promote caspase-dependent endogenous apoptosis pathway, thus inhibiting cancer cells [40]. Hyperoside can promote the dissociation of Nrf2 from the keap-1/Nrf2 complex, and thereafter promote the entrance of the dissociated Nrf2 into the nucleus, where it induces the expression of Nrf2-encoded antioxidants proteases and regulates ROS level in the body. In the same way, it can also regulate the apoptosis of cancer cells [41]. Astragalin can reduce inflammation and regulate the process of oxidative stress [42], possibly endowing it with the ability to induce apoptosis of cancer cells. A study on phloridzin has shown that it has antitumor activity, especially when treating skin cancer, and this effect is mainly due to its regulation of oxidative stress [43]. Quercetin can inhibit the growth of cancer cells in vitro and the RNA and proteins [44]. These five bioactive flavonoids constitute that main components in LLF. Studies have shown that kaempferitrin can regulate the expression of caspases, and has a certain target effect on inflammation TLR4, TNF-α, NLRP3, caspase-1, IL-18, IL-1β, SREBP-1c, PPAR-γ and UCP2 [45], hyperoside, astragalin and quercetin inhibits inflammatory responses in vitro via p38 pathways [46–48], and phloridzin also showed the effect of regulating p38 in vivo to inhibit inflammation [49]. LLF contains these five components, so it also shows the regulation of caspases and p38. Due to their combined effect, LLF has promoted apoptosis of cancer cells through the ROS/p38 MAPK regulatory pathway (Fig. 11).

## Conclusions

In this study, we investigated the apoptosis-inducing effect of LLF in A549 lung cancer cells in vitro. Results show that LLF is nontoxic to normal cells up to a certain concentration, but could inhibit the proliferation of A549 lung cancer cells. LLF could induce apoptosis of A549 cells in concentrations that were deemed safe for normal cells. It was further shown that LLF could affect genes related to the ROS/p38 MAPK pathway, so as to exerted its effect on proliferation inhibition and apoptosis induction of A549 cells. LLF are composed of five main component flavonoids that are the main enhancers of cancer cells inhibition. It is, therefore, clear that LLF, through its active components, can inhibit cancer cells in vitro. The results of this study provide a theoretical basis for future studies of LLF.

## Data Availability

All the data generated or analyzed during this study is available.

## Abbreviations

LLF: Lotus leaf flavonoids
ROS: Reactive oxygen species
MDA: Malondialdehyde
SOD: Superoxide dismutase
qRT-PCR: Quantitative real-time polymerase chain reaction
Bax: Bcl2-associated X protein
Bcl-2: B-cell lymphoma-2
Cu/Zn SOD: Copper/zinc superoxide dismutase
CAT: Catalase
Nrf2: Nuclear factor erythroid-2 related factor 2
NQO1: NAD(P)H dehydrogenase [quinone] 1
HO-1: Heme oxygenase-1
HPLC: High performance liquid chromatography
